# Electromyography-guided percutaneous needle electrolysis for masticatory myofascial pain: A prospective clinical study

**DOI:** 10.4317/jced.64550

**Published:** 2026-08-28

**Authors:** Luis Miguel Gonzalez-Perez, Ramon Vera-Martin, Jose Francisco Montes-Carmona, Enrique Montes-Latorre, Pedro Infante-Cossio

**Affiliations:** 1 Department of Oral and Maxillofacial Surgery, Virgen del Rocio University Hospital, Seville, Spain; 2 2 Department of Surgery, School of Medicine, University of Seville, Seville, Spain; 3 Department of Clinical Neurophysiology, Virgen del Rocio University Hospital, Seville, Spain

**Keywords:** Temporomandibular disorders, myofascial pain, trigger points, percutaneous electrolysis, electromyography.

## Abstract

**Background:**

Masticatory myofascial pain syndrome (MMPS) is characterized by the presence of trigger points (TrPs) in the masticatory musculature. Percutaneous needle electrolysis (PNE) is a minimally invasive technique with proven efficacy in tendinopathies, although its application in the temporomandibular region is limited. The objective was to evaluate the clinical effectiveness of a single session of electromyography (EMG)-guided PNE on pain, function, and mandibular mobility in patients with MMPS.

**Materials and Methods:**

A prospective clinical study was designed involving 26 patients diagnosed with MMPS with active TrPs (80.8% women). A single session of PNE was administered to the masticatory musculature under EMG monitoring. Pain intensity (visual analogue scale), mandibular mobility (maximum interincisal opening, lateral and protrusive excursions), and temporomandibular joint (TMJ) function were evaluated using a validated questionnaire. Measurements were obtained at baseline and at 7, 30, 90, and 180 days post-treatment. Non-parametric tests were performed for statistical analysis.

**Results:**

A statistically significant reduction in pain intensity was observed from the first week post-intervention (VAS score: 6.64 vs. 2.76; p<0.001), which remained sustained throughout the 6-month follow-up. The global functional assessment of the TMJ and all ranges of mandibular mobility showed significant early increases (day 7), which remained unaltered and clinically meaningful until the end of the study. Mild and self-limiting adverse effects were experienced by 11.5% of the patients.

**Conclusions:**

A single session of EMG-guided PNE constitutes a useful therapeutic alternative for the management of MMPS, as it provides early and sustained pain reduction, along with a significant restoration of mandibular dynamics and function. Controlled comparative studies are needed before its efficacy can be definitively established.

## Introduction

Masticatory myofascial pain syndrome (MMPS) is one of the leading causes of non-inflammatory craniofacial pain. It constitutes a common reason for consultation among patients with temporomandibular disorders (TMDs), which include a heterogeneous group of musculoskeletal and articular conditions affecting the masticatory system [1]. According to the Diagnostic Criteria for Temporomandibular Disorders (DC/TMD), myogenous TMDs comprise local myalgia and myofascial pain with or without referral are found [2], representing the most frequent phenotypes in clinical practice. MMPS is clinically characterized by the presence of taut muscle bands containing myofascial trigger points (TrPs) in the form of hyperirritable nodules, the palpation of which elicits regional or referred pain, altered motor activity, and restriction of mandibular movements [1-4]. In the masticatory system, symptoms are most frequently attributed to the masseter, temporalis, and pterygoid muscles. The painful condition is often accompanied by functional limitations and autonomic or dysautonomic phenomena [5].

From an epidemiological perspective, TMDs may affect a high percentage of the population when subclinical cases and transient or chronic forms are included [5]. They show a clear predilection for females (a threefold higher risk) and a peak incidence between 18 and 44 years of age [6]. An accurate diagnosis is primarily based on a standardized medical history and physical examination, which can be complemented, when necessary, by imaging and neurophysiological techniques to rule out other causes of pain or mechanical joint limitation [4].

The etiopathogenesis of MMPS is multifactorial and complex, involving predisposing, initiating, and perpetuating factors. These include sustained microtrauma (such as bruxism), persistent nociceptive input arising from structures outside the temporomandibular joint (TMJ), and psychological factors, such as anxiety and depression, which sensitize afferent pathways and lower the pain threshold [7]. Among TMDs, myofascial pain is the most frequent diagnosis and concentrates the greatest burden of risk factors [5]. Given this heterogeneity, there is no definitive therapeutic consensus regarding its treatment. Management must be individualized and stepped, beginning with patient education, behavioural modification, and the administration of analgesic and anti-inflammatory drugs. Subsequently, conservative and non-invasive modalities are employed (occlusal appliances, manual physiotherapy, or pharmacologic measures); however, a significant percentage of patients are refractory to these treatments [8,9]. For these challenging cases, invasive interventional therapies aimed at the mechanical disruption of TrPs have been proposed, such as dry needling or the infiltration of substances (local anaesthetics or botulinum toxin). Nevertheless, clinical evidence continues to show variability regarding their long-term success rates [10-14].

In recent years, percutaneous needle electrolysis (PNE) has emerged as a minimally invasive alternative that involves applying a continuous galvanic electrical current directly to the TrP through an acupuncture needle, which induces controlled tissue necrosis and activates intramuscular regeneration mechanisms [15]. Although its efficacy has been widely documented in tendinopathies and muscular fasciitis, its application in MMPS is still in its early stages and lacks standardized protocols [15,16]. To maximize diagnostic and therapeutic precision, the incorporation of needle electromyographic (EMG) guidance allows for the monitoring of the spontaneous electrical activity of the active locus of the TrP at rest and during contraction [17]. Neurophysiological guidance aims to improve anatomical localization and precise entry into the affected muscle belly, facilitating access to deep and difficult-to-reach muscles, such as the lateral pterygoid, and minimizing the risk of adverse effects.

The objective of this study was to evaluate the clinical effectiveness, time course, and safety profile of the EMG-guided PNE technique for the treatment of patients with MMPS. It is hypothesized that a single, low-intensity session in the masticatory musculature can significantly and sustainably reduce pain intensity and optimize mandibular dynamics, with a minimal incidence of mild complications.

## Materials and Methods

*Study design and patients*

A prospective and longitudinal clinical study was designed to evaluate the effectiveness and safety of EMG-guided PNE in patients diagnosed with MMPS with active TrPs. Recruitment was carried out at the Department of Oral and Maxillofacial Surgery of the Virgen del Rocio University Hospital (Seville, Spain) over a 12-month period (from January 2022 to January 2023).

The sample size was calculated a priori using pain intensity, measured by the visual analogue scale (VAS), as the primary outcome. A minimum reduction of 2 points from the baseline value was considered clinically relevant. Assuming a standard deviation of 2.5 points, a two-tailed alpha error of 5%, and a statistical power of 80%, a minimum sample size of 24 patients was estimated. Considering a potential dropout rate of 10% during follow-up, the final required sample size was 26 subjects. Out of a total of 36 patients initially assessed in the pre-inclusion consultation, 28 ultimately met the selection criteria and were included in the study.

*Selection criteria*

The inclusion criteria were as follows: Patients aged between 18 and 75 years with predominantly myogenous pain in the temporomandibular region, a symptom duration of between 6 and 12 months, and the presence of active TrPs in the masticatory musculature, according to DC/TMD examination procedures.

Patients presenting any of the following conditions were excluded: Severe limitation of mandibular mobility, internal joint derangement or degenerative pathology confirmed by imaging, infectious or inflammatory processes in the needling site, history of mandibular fractures, degenerative neuromuscular diseases, coagulation disorders or anticoagulant treatment, fibromyalgia, major depression, uncontrolled metabolic disease, needle phobia, refusal to participate, or pregnant or breastfeeding.

*Ethical considerations*

The study was approved by the Research Ethics Committee of the Virgen del Rocio University Hospital (IRB: PI 16/970) and was conducted in accordance with the Declaration of Helsinki. Participants were duly informed of the objectives, risks, and benefits of the intervention and provided their written informed consent prior to inclusion.

*Clinical protocol and intervention*

In the initial assessment, a comprehensive medical history and a standardized physical examination of the masticatory musculature (masseter, temporalis, and medial and lateral pterygoid muscles) were performed following a systematized protocol of palpation, pressure, pincer grasp, and sliding to locate painful taut bands and TrPs. Additionally, imaging studies (orthopantomography and magnetic resonance imaging) were requested to rule out underlying joint pathology.

The treatment was administered in a single session (day 0) on the muscles presenting clinically relevant TrPs. Prior to the puncture, an EMG study was performed at the Clinical Neurophysiology Department to accurately locate and record the activity patterns of the involved muscle groups, both at rest and during voluntary contraction (Figure 1A). After skin disinfection with 2% chlorhexidine, the PNE technique was applied individually to the affected muscles. Acupuncture needles (0.30×40 mm) were inserted into the intramuscular TrP, and a 0.1 mA galvanic current was applied for 1 minute (Figure 1B). Subsequently, manual haemostatic compression was applied for 2-3 minutes, and a re-examination was performed to intervene on any other untreated satellite nodule, if clinically indicated, during the same procedure.

*Study variables and follow-up*

The primary study outcome was pain intensity, measured using the VAS ranging from 0 (without pain) to 10 (worst pain imaginable). Secondary variables included the range of mandibular mobility (maximum interincisal opening, lateral and protrusive excursions, in mm) and the global functional assessment of the TMJ using a validated 100-point questionnaire (0 worst case, 100 optimal), based on pain in daily activities (maximum 40 points), function (45 points), and mastication (15 points). Clinical follow-up lasted for 6 months, with variables recorded at the baseline visit and at subsequent visits at 7, 30, 90, and 180 days post-intervention. Side effects were also recorded. All clinical assessments and the therapeutic procedure were performed by the same operator in order to minimize inter-examiner variability.

*Statistical analysis*

Statistical data analysis was performed using the IBM SPSS Statistics software. A per-protocol analysis was performed to evaluate the evolution of pain, mandibular mobility, and TMJ function throughout the follow-up period and was compared with pre-treatment values. Since the variables did not meet the assumptions of normality (Shapiro-Wilk test), non-parametric analyses were performed. To determine the statistical significance of overall changes over time, the non-parametric Friedman test (χ²) was used. A Wilcoxon signed-rank test was performed to make pairwise comparisons between the initial value and each subsequent checkpoint, as well as between consecutive checkpoints. Effect sizes were expressed as Kendall’s W for Friedman tests and r for Wilcoxon comparisons. Values of *p<*0.05 were considered to indicate statistical significance. When the Bonferroni correction was applied for multiple comparisons, the statistical significance was *p<*0.008.

## Results

Initially, 28 patients were included in the study, of whom 26 completed the 6-month follow-up period and constituted the population for the per-protocol statistical analysis. Two patients withdrew from the study due to a change of residence and failure to attend follow-up appointments. The analysed sample showed a clear female predominance (80.8%), with a mean age of 43.39 ± 14.92 years (Table 1).

The primary outcome (pain intensity measured by VAS) showed a statistically significant reduction throughout the follow-up period (Friedman test: χ²=71.80; *p<*0.001), with a Kendall's W effect size of 0.69. Comparisons using the Wilcoxon signed-rank test with Bonferroni post hoc correction revealed a significant pain reduction between the baseline assessment and the first week post-treatment (Z=-4.51; *p<*0.001; r=0.88). This was reflected in a clinically relevant decrease of -3.88 points on VAS in the first week, decreasing from a baseline score of 6.64 ± 1.07 to 2.76 ± 1.01. This analgesic improvement remained clinically stable with no significant differences at the 30-, 90-, and 180-day follow-up points (Table 2, Figure 2).

Maximum interincisal opening showed significant differences during the follow-up period (χ²=42.42; *p<*0.001; W=0.40). The initial improvement was 3.08 mm during the first week (Z=-3.63; *p<*0.001; r=0.71), with significant additional increases observed between days 7 and 30 (*p<*0.001) and between days 30 and 90 (*p=*0.005) that remained significant after the Bonferroni correction, stabilizing at an average of 42.12 ± 3.92 mm at 6 months (Table 2, Figure 3A).

Right (χ²=31.01; W=0.29) and left (χ²=23.26; W=0.22) lateral excursion movements also improved significantly (*p<*0.001 in both cases), with increases of 1.74 mm and 1.31 mm, respectively, during the first week post-intervention. They remained unaltered and close to 7 mm at subsequent follow-up visits. Protrusive excursion showed an overall significant improvement (χ²=49.60; W=0.47; *p<*0.001), with increases of 1.21 mm during the first week and 0.73 mm up to the first month, remaining stable thereafter (Table 2, Figure 3B).

The global functional assessment of the TMJ showed the largest effect size among all the analysed variables (χ²=86.51; W=0.83; *p<*0.001). The score increased by a mean of 15.11 points during the first week (Z=-4.48; *p<*0.001; r=0.88), achieving functional stability of the TMJ at around 85 points until the end of the study period (Table 2, Figure 4).

The magnitude of the changes observed between the baseline assessment and the 6-month follow-up showed a mean pain reduction of 4.04 points (95% CI -4.9 to -3.4), accompanied by a mean increase of 4.28 mm in maximum interincisal opening (95% CI 3.6 to 6.6). Likewise, improvements were observed in right (increase: 2.01; 95% CI 1.3 to 3.7) and left (increase: 1.59; 95% CI 0.9 to 3.1) lateral excursion, protrusive excursion (increase: 1.94; 95% CI 1.0 to 3.0), and TMJ functional assessment (increase: 15.92; 95% CI 13 to 23).

Regarding the safety of the procedure, the overall incidence of side effects was 11.5% (n=3). Only minor and self-limiting complications associated with the puncture site were recorded: One case of post-puncture hematoma and two cases of transient local pain, which were managed conservatively and satisfactorily controlled with first-line analgesics. No adverse events affected the patients' activities of daily living or led to their withdrawal from the study.

## Discussion

This prospective and longitudinal study evaluated the clinical effectiveness and safety profile of a single application of EMG-guided PNE in patients with MMPS. The main findings revealed a clinical and statistically significant improvement in pain intensity, mandibular kinematics, and TMJ functionality. These improvements became evident early on, from the first week of treatment, and remained unaltered throughout the 6-month follow-up period, supporting the hypothesis that PNE could represent a valid and minimally invasive therapeutic alternative for this population. This pattern of early improvement and subsequent stabilization highlights the initial therapeutic effect of this technique, rather than a progressive or late long-term change. In addition to the statistical significance, the magnitude of the changes recorded at 6 months is of clinical relevance. The mean pain reduction reached 4.04 points on the VAS, exceeding the minimum threshold considered clinically important for patients with TMDs. Similarly, the sustained functional improvement over the 6-month follow-up, reflected in the mean increase in interincisal opening and in lateral and protrusive excursion movements, reinforces the clinical relevance of the treatment, as these parameters are directly related to the patient's quality of life.

MMPS is a debilitating condition that has a considerable adverse impact on patients' social and occupational lives, as well as on their quality of life, representing one of the one of the most common causes of consultation for craniofacial pain [1,18]. The profile of our sample, with a female predominance and a mean age in the fourth decade of life, is consistent with the known epidemiology of TMDs [7]. Despite its high prevalence, clinical management lacks a unified consensus, largely due to the fact that a significant percentage of cases exhibit refractoriness to traditional conservative modalities such as occlusal appliances, physiotherapy, or pharmacotherapy [8,9]. An alternative to these classic conservative approaches is therapies based on muscle needling techniques [19]. In this context, minimally invasive treatments become particularly relevant.

The findings of this study support the clinical use of PNE as a useful tool in refractory cases, as it was associated with sustained reductions over time of more than 3 points on VAS. Our results are consistent with previous studies describing the beneficial effects of PNE in musculoskeletal conditions, although specific evidence in the orofacial region remains limited and heterogeneous. The systematic review by Trybulski *et al*. [20] noted that PNE can reduce pain and improve functional parameters in MMPS, with a favourable safety profile and minimal, self-limiting adverse effects. However, the authors warn that these findings should be interpreted with caution due to the latent risk of bias in most of the literature, stemming from the methodological difficulties in achieving blinding of control groups.

The results of the present study support our previous findings reported in a double-blind randomized clinical trial [21]. In that pioneering study, the application of PNE already demonstrated a significant reduction in pain and functional improvement superior to placebo, with an earlier response than that obtained by conventional dry needling, without recording any severe adverse effects. Similarly, Gonzalez-Perez *et al*. [17] demonstrated in a randomized clinical trial that the application of PNE constitutes a therapeutic alternative as valid and safe as the injection of botulinum toxin type A for the treatment of refractory chronic masticatory myalgia. In that study, both interventions showed high efficacy in reducing pain and improving muscle function, achieving an improvement that persisted unaltered over a three-month period. The data from our current prospective cohort corroborate this favourable therapeutic response and its low profile of adverse effects. They also suggest that the clinical benefit of a single PNE session can extend up to the 6-month follow-up.

Unlike conventional PNE for tendinopathies, which uses high intensities (1-3 mA) in order to achieve tissue ablation, the management of myofascial pain requires a better-tolerated dosage. In our study, we applied 0.1 mA for 1 minute per TrP to prioritize neuromodulation over structural destruction. The meta-analysis published by De la Barra Ortiz *et al*. [22] found that, although all electrolysis modalities significantly reduce pain intensity and musculoskeletal disability, the use of microelectrolysis (application of currents in the range of 100 to 990 µA) achieves an analgesic effect size even superior to that of higher intensities. Furthermore, a single PNE session seems capable of activating endogenous pain modulation mechanisms, achieving similar analgesic effects while ensuring patient tolerance and clinical comfort [23]. This microelectrolysis regimen is particularly indicated in the complex masticatory musculature, as it minimizes nociceptive stimulus, avoids reflex muscle spasm, and prevents iatrogenic effects in hypersensitive patients [10,15-18,21,24,25]. In addition, the administration of galvanic current through the needle could combine the mechanical effect of TrP needling with a local electrochemical stimulus that promotes the dissociation of the dysfunctional locus and subsequent tissue remodelling. The biological basis of this tissue remodelling is grounded in the controlled pro-inflammatory response and cellular apoptosis during the first seven days, which would eliminate dysfunctional cells, creating a favourable environment for extracellular matrix synthesis and subsequent healing [26]. 

With the introduction of the DC/TMD diagnostic criteria in 2014, pain was consolidated as the central symptom of TMDs, replacing the concept of myofascial pain syndrome with specific diagnoses such as local myalgia [2]. For the selection and homogenization of the sample, we classified the condition within the category of local or referred masticatory myalgia [2,24]. This methodological delimitation is crucial, as the presence of pain circumscribed to the masticatory musculature and the precise identification by palpation of the hypersensitive TrP nodule, which caused strictly localized pain without radiation, were the indications for treatment in our study [27]. The strict anatomical delimitation avoided the inclusion of conditions with extracranial irradiation or of purely articular origin, which allowed the effect of the therapy on the myogenic tissue to be isolated.

One of the main technical contributions of this protocol was the simultaneous EMG guidance during the application of galvanic current. Although previous studies have analysed the use of PNE in musculoskeletal conditions, the literature focusing on the orofacial region is scarce and heterogeneous in terms of variables and protocols [15-17,21]. Monitoring via EMG allowed for the precise localization of the target muscle groups and the identification of involvement patterns at rest and during contraction. It is postulated that this spatial precision optimizes analgesic efficacy by focusing the microcurrent directly on the TrP of the affected muscle, which allows for the use of the minimum necessary dose and exposure time, translating directly into a reduced risk of iatrogenic injury or adverse side effects [17].

The results of the present study are consistent with those of previous minimally invasive studies conducted by our group and others that investigated dry needling, botulinum toxin, and percutaneous electrolysis in masticatory myalgia [7,8,11-14,28]. However, it is important to note that comparisons between studies should be made with caution. Eligibility criteria, diagnostic frameworks, follow-up periods, and outcome assessment instruments vary substantially in the TMD literature. Even when studies nominally evaluate similar interventions, differences in patient phenotype (local myalgia, referred myofascial pain, intra-articular comorbidity, or parafunctional load) can significantly influence the response.

The observed safety profile was very favourable. The rate of minor side effects (11.5%) was exclusively related to the puncture site (local hematoma and transient self-limiting pain), with no major or disabling adverse events recorded. This excellent tolerance, together with the significant increase in the TMJ functional questionnaire score, reinforces the viability of the technique in the clinical setting.

However, this study presents methodological limitations that should be considered when interpreting the results. Due to its single-cohort observational design, without an active control or placebo group, the data allow for the establishment of robust statistical associations of improvement, but not a direct and unequivocal causal relationship. Regression to the mean, the natural fluctuation of symptoms, contextual effects, and co-interventions cannot be ruled out. Furthermore, the small sample size and single-centre design limit the generalizability of the data (external validity), and the 6-month follow-up period precludes documenting long-term recurrence rates. Finally, psychosocial variables such as anxiety or depression, widely recognized factors that modulate pain chronicity in TMDs [11], were not included in the formal analysis.

Consequently, although current literature does not establish a clear superiority among the various interventional therapies for TMD of myogenic origin, the use of EMG-guided PNE emerges as an option with a favourable risk-benefit ratio. This study should be understood as a prospective, hypothesis-generating cohort study. The results obtained are sufficient to justify conducting future multicentre, randomized, placebo-controlled clinical trials (sham design) with longer follow-up periods to standardize protocols in the orofacial region. The systematic review by Pirri *et al*. [29] identified PNE as an effective tool for reducing musculoskeletal pain but emphasized the need to standardize clinical parameters and validate the technique through rigorous cost-effectiveness studies before recommending it as a first-line treatment. Furthermore, conducting a complementary cost-effectiveness analysis would provide key information to evaluate the feasibility of incorporating this technique into health services.

## Conclusions

The application of a single session of low-intensity EMG-guided PNE in patients with MMPS appears to be a useful, safe and minimally invasive therapeutic option. This procedure significantly reduces pain intensity and improves TMJ function and ranges of mandibular dynamics (maximum interincisal opening, lateral and protrusive excursions). These clinical benefits are evident from the first week post-treatment and remain unaltered over a 6-month follow-up period. Furthermore, the technique presents a favourable safety profile, with infrequent, mild, and self-limiting adverse effects. Given that the study design does not allow for establishing conclusive causal relationships, these results should be interpreted as supporting the feasibility and potential benefit of the procedure, rather than as definitive proof of its efficacy. Comparative controlled studies are warranted. 

## Figures and Tables

**Table 1:** Baseline demographic characteristics (n=26).

**Variable**	**Value**
Gender	
Females, n (%)	21 (80.8%)
Males, n (%)	5 (19.2%)
Age (years), mean ± SD (median)	43.39 ± 14.92 (41)

**Table 2:** Baseline demographic characteristics (n=26).

	**Baseline (Day 0)¹**	**Day 7¹**	**Day 30¹**	**Day 90¹**	**Day 180¹**	**Overall p-value²**
Pain (VAS)	6.64 ± 1.07 (7)	2.76 ± 1.01 (3)*	2.52 ± 1.23 (2)	2.60 ± 1.35 (2)	2.60 ± 1.35 (2)	<0.001
MIO (mm)	37.84 ± 5.65 (35)	40.92 ± 4.91 (40)*	41.64 ± 4.47 (40)†	42.06 ± 4.28 (40)‡	42.12 ± 3.92 (41)	<0.001
Right laterotrusion (mm)	5.07 ± 1.68 (5)	6.81 ± 1.79 (7)*	6.96 ± 1.82 (7)	7.00 ± 1.70 (7)	7.08 ± 1.62 (7)	<0.001
Left laterotrusion (mm)	5.50 ± 1.40 (5)	6.81 ± 1.81 (6.5)*	7.00 ± 1.96 (6.5)	7.15 ± 2.13 (6.5)	7.00 ± 1.70 (6.5)	<0.001
Protrusion (mm)	5.14 ± 1.58 (5)	6.35 ± 1.72 (6)*	7.08 ± 1.60 (7)†	7.12 ± 1.68 (7)	7.08 ± 1.41 (7)	<0.001
TMJ functional score	68.93 ± 15.30 (67)	84.04 ± 15.85 (90)*	84.58 ± 15.10 (90)	85.12 ± 14.28 (90)	84.85 ± 13.63 (90)	<0.001

^1^ Values are expressed as mean ± SD (median). ^2^ Overall significance test using the Friedman test (χ^2^). * Statistically significant difference compared to baseline (p<0.008 adjusted by Bonferroni post hoc correction). † Statistically significant difference compared to day 7 (p<0.008 adjusted by Bonferroni post hoc correction). ‡ Statistically significant difference compared to day 30 (p<0.008 adjusted by Bonferroni post hoc correction).

**Figure 1:**
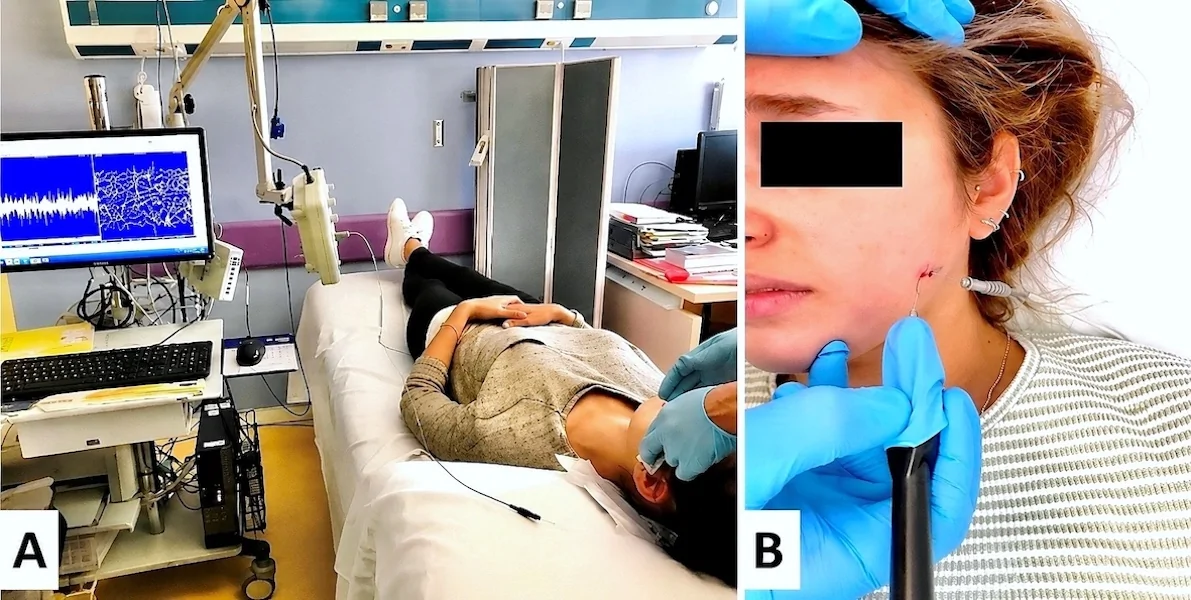
A: Localization of the TrP in the masseter muscle using EMG. B: Insertion of the PNE under EMG guidance.

**Figure 2:**
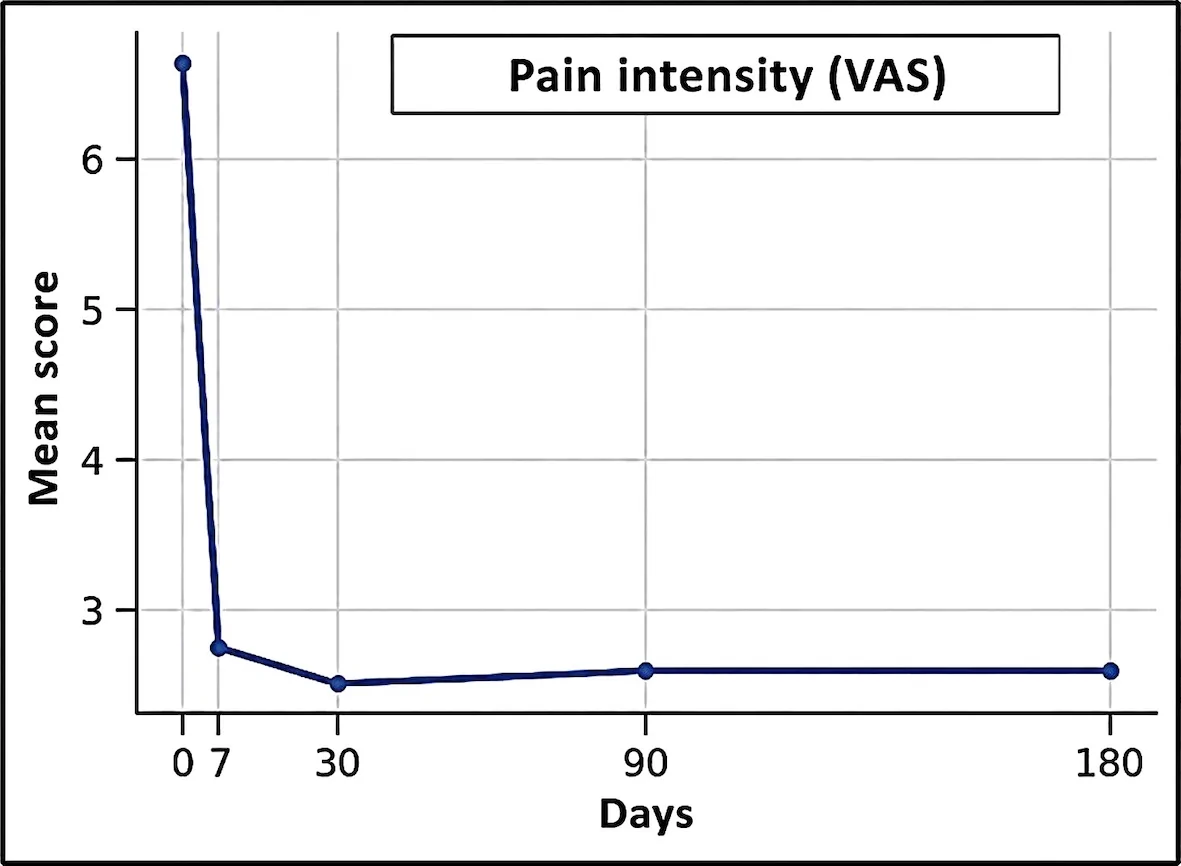
Pain intensity at 6 months.

**Figure 3:**
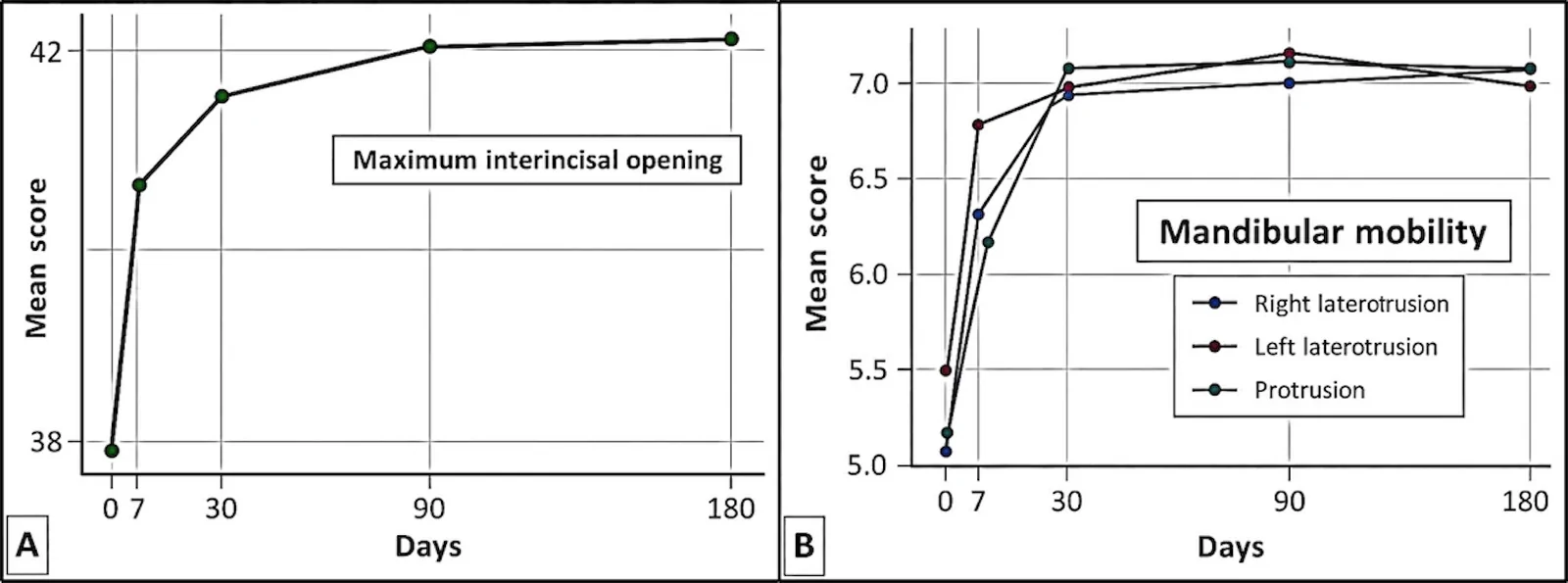
A: Maximum interincisal opening at 6 months. B: Mandibular mobility at 6 months.

**Figure 4:**
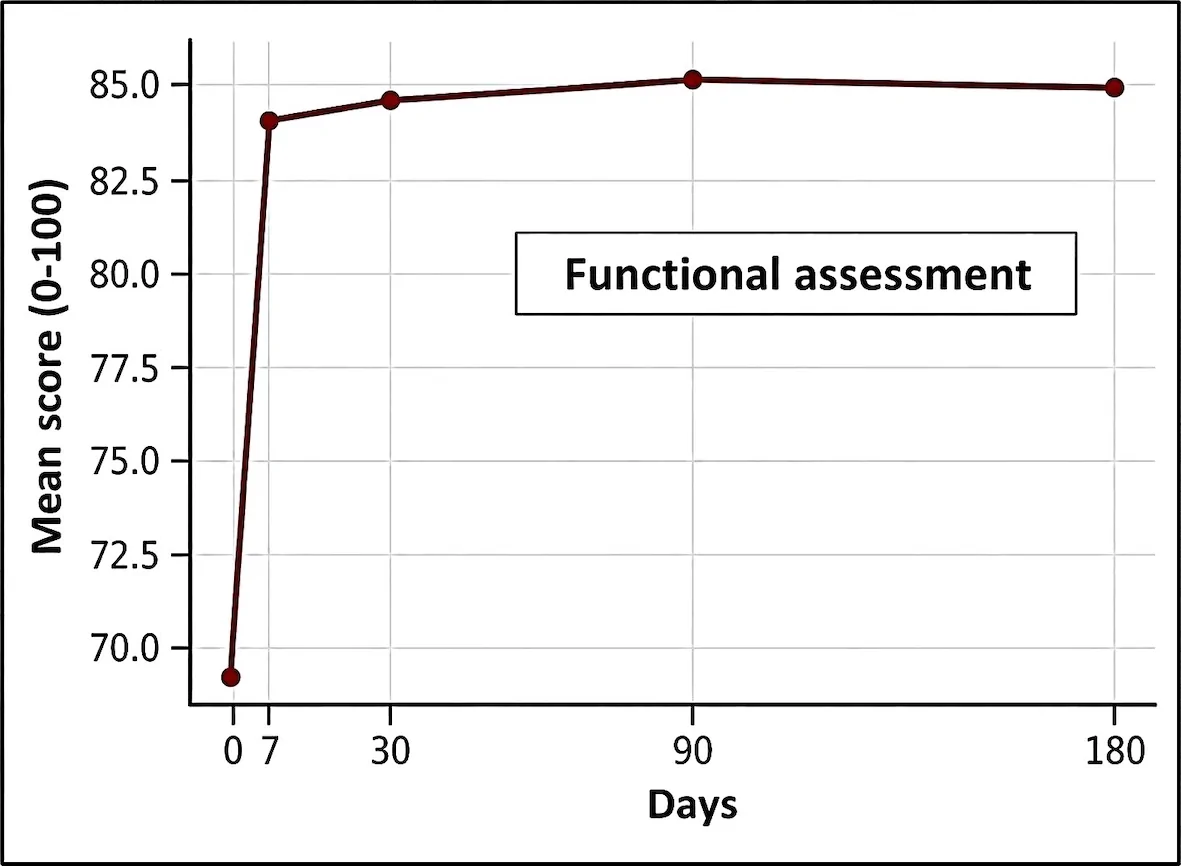
TMJ functional assessment at 6 months.

## Data Availability

Declared none.
